# TRPM2 facilitates tumor progression of clear cell renal cell carcinoma by relieving Endoplasmic Reticulum Stress

**DOI:** 10.7150/ijms.77944

**Published:** 2023-01-01

**Authors:** Hongwei Yuan, Weiquan Li, Ning Lou, Tiexi Yu, Xiangui Meng, Wen Xiao, Xiaoping Zhang

**Affiliations:** 1 Department of Urology, Union Hospital, Tongji Medical College, Huazhong University of Science and Technology, Wuhan 430022, China.; 2 Department of Urology, The Central Hospital of Wuhan, Tongji Medical College, Huazhong University of Science and Technology, Wuhan, China.

**Keywords:** TRPM2, Endoplasmic Reticulum Stress, ccRCC, Tumor progression

## Abstract

Clear cell renal cell carcinoma (ccRCC) has the highest incidence rate among all pathological types of kidney cancers. Although the role of transient receptor potential (TRP) ion channel TRPM2 has been studied in many cancers, its function in ccRCC is still unexplored. In this study, using the KIRC module of TCGA, we found that TRPM2 was upregulated in ccRCC tissues and was related to poor prognosis. Gene set enrichment analysis (GSEA) showed that TRPM2 was related to epithelial-to-mesenchymal transition (EMT), TCA cycle, fatty acid metabolism, and immune system-related functions. Functional experimental results indicated that TRPM2 could promote ccRCC progression. Furthermore, mechanism analysis showed that knocking out TRPM2 can reverse these phenotypes by increasing endoplasmic reticulum stress and decreasing EMT. We also investigated the potential role of TRPM2 in immune cell infiltration in the tumor microenvironment. Our study indicated that TRPM2 promotes ccRCC progression and may be a novel target for ccRCC therapy.

## Introduction

Renal cell carcinoma is one of the most common cancers. Approximately 76,080 new cases and 13,780 deaths have been reported in the USA in 2021 1. About 75% of all pathological subtypes of RCC are clear cell renal cell carcinoma (ccRCC) 2, and it is closely related to a mutation in the gene named von Hippel-Lindau (VHL) 3. For a long time, the most important treatment for RCC has been surgery and chemotherapy 4, but the prognosis of surgical resection for advanced RCC is unsatisfactory. It is reported that tumors in around 30% of patients recur or metastasize after surgery 5. In recent years, targeted drug therapy has offered effective treatment with receptor tyrosine kinase inhibitors like sunitinib 6. However, patients undergoing drug therapy for a long time could develop drug resistance 7, 8. Therefore, it is of great urgency to explore more effective biomarkers and therapeutic targets that may improve the survival rate of ccRCC.

The functions of transient receptor potential (TRP) ion channels in biological processes and diseases have attracted increasing attention 9, 10. The TRPM subfamily, which includes 8 members 11, TRPM1-TRPM8, is a subgroup of TRP channel superfamily. Transient receptor potential (TRP) melastatin 2 (TRPM2), as a cation channel, allows Ca^2+^ to penetrate cell membrane 12. The C-terminal NUDT9H domain in human TRPM2 hydrolyzes adenosine diphosphate ribose (ADPR) 12. Studies have shown that ADPR and oxidative stress activate human TRPM2 13, 14. Although TRPM2 has emerged as a potential therapeutic target in various cancers 15-17, its exact role in ccRCC is still unclear.

The endoplasmic reticulum (ER), the largest organelle in eukaryotic cells, participates in several cellular biological process including calcium balance and synthesis of proteins and phospholipids 18. Nevertheless, increasing research shows that ER is involved in some pathological processes, among which, ER stress attracted people's interest 19. Under stress conditions, the ER environment is abnormal, and protein formation is impaired, causing unfolded protein response (UPR) and accumulating abnormal proteins 18. Subsequently, three primary ER stress sensors, protein kinase RNA (PKR)-like endoplasmic reticulum kinase (PERK), inositol-requiring enzyme 1α (IRE1α), and activating transcription factor 6 (ATF6), initiate UPR signaling and adaptive processes 20. BiP, one of the most abundant proteins in the endoplasmic reticulum, is altered from chaperone to ER stress sensor by IRE1 and PERK 21. Stdudies have shown that ER homeostasis can protect cancer cells 22, while high-intensity ER stress may initiate cell decease via UPR 23.

The phenomenon of epithelial-mesenchymal transition (EMT) was initially found in chick embryos, which played a vital role in embryonic development 24. Later research showed during this process, epithelial cells gradually lost adhesion and junctions between cells 25, 26. EMT is essential to promote cancer progression and metastasis. And EMT biomarkers E-cadherin (CDH1) and cytokeratins are negatively correlated, while N-cadherin (CDH2), vimentin (VIM) and Zinc finger protein SNAI1 (SNAIL) show a positive correlation with EMT 27.

Here, we explored the expression profile of TRPM2 using the clinical and pathological data in the KIRC module of TCGA database. Gene set enrichment analysis (GSEA) and DAVID was used to explore the biological role of TRPM2 in ccRCC. It was discovered that TRPM2 was upregulated in ccRCC and could be a candidate biomarker of ccRCC and can predict prognosis. *In vitro* experiments showed TRPM2 enhanced malignant potential of tumor cells. Mechanism assays showed TRPM2 reduction enhanced ER stress and reduced EMT. We also found that TRPM2 is closely associated with infiltration of immune cells. Therefore, our study offers a possible therapeutic target for ccRCC.

## Materials and methods

### Patient tissue samples

All matched tumor and para-tumor tissues were obtained from patients in the Department of Urology, Wuhan Union Hospital, from 2019-2020. These patients underwent renal tumor surgery, and postoperative pathological findings confirmed ccRCC. Our study followed the Declaration of Helsinki and was approved by the Ethics Committee of Human Research of Huazhong University of Science and Technology.

### Cell culture

Human RCC cell lines (CAKI, 786-O, A498, OSRC, and ACHN) and corresponding normal cell line HK-2 were acquired from the American Type Culture Collection (ATCC) and cultured according to the standard protocol.

### RNA extraction and qRT-PCR

The MagZol reagent (Magen, Shanghai, China) was used to obtain RNA, following the manufacturer's protocols. The RNA concentration was determined by NanoDrop 2000 spectrophotometer (NanoDrop Technologies, USA). Reverse transcription was conducted according to protocol. qPCR analysis was performed following standard protocols. All assays have been repeated triplicated. All samples were normalized using β-actin. Primer sequences are listed below:

- β-actin: forward 5′-TCACCATGGATGATGATATCGC-3′; reverse 5′- ACATAGGAATCCTTCTGACCCA -3′.
- TRPM2: forward 5′-TTCGTGGATTCCTGAAAACATCA-3′; reverse 5′-CCAGCATCAGACAGTTTGGAAC-3′.

### Western Blotting

RIPA buffer (Beyotime, China) with cocktail and PMSF was used to lyse the cells and extract protein. SDS-PAGE gel at 120V was used to separate the protein sample, and the protein blots were transferred to the membranes at 250 mA for 90 min. These membranes were blocked in 5% non-fat skimmed milk for 90 min at room temperature. Then the membranes were incubated overnight with primary antibodies at 4 °C. The following primary antibodies were used: ER Stress Antibody Sampler Kit #9956 (BIP, ERO, IREB), TRPM2 (1:1000; Abclonal, A6137), snail (1:1000; Abclonal, A5243), N-cadherin (1:1000; Abclonal, A0433), Vim (1:1000; Abclonal, A2584), E-cadherin (1:1000; Abclonal, A11509), and β-actin (1:1000; Proteintech, 20536-1-AP). After being washed and incubated with secondary antibodies, the membranes were developed to detect bands. ImageJ was used to conduct quantification of Western blots.

### Cell transfection

The SYNTHEGO (https://design.synthego.com/#/) was used to design the sgRNA sequences. The plasmids were constructed and purchased from GeneRulor (Wuhan, China). Lipofectamine 3000 reagent (Invitrogen by Thermo Fisher Scientific, Carlsbad, CA) was used for plasmids transfection following the manufacturer's instructions when cells were 70-80% confluent, with 3 ug plasmids (sg-NC or sg-TRPM2) per well (6-well culture plate). After incubation for 1-3 days, cells were collected to evaluate the transfection efficiency via qRT-PCR or Western blot. sgRNA sequences are as follows:

- sg-TRPM2-1, 5′-GCUGUGUCUUUGCAGGAAGG-3′;
- sg-TRPM2-2, 5′-AGGAAGGUGGUGUGUCAGUG-3′;
- sg-TRPM2-3, 5′-CUACACGCAUGAGCAGCACU-3′.

### Cell functional assays

After successfully introducing sg-TRPM2 and sgRNA negative control (NC) plasmids into cells, the 786-O and A498 cells were plated on the 96-well plate (2,000/well) for the proliferation assay. The proliferation rate was measured by CCK8 every alternate day for 4 days. The transwell assay was conducted as described previously 28. For migration assays, cells were plated with a density of 3×10^4^/chamber; for invasion assays, the number was double. For colony formation assays, 1000 cells were plated per well (6-well culture plates), and after being cultured for about 2 weeks, cells were fixed with methanol and stained with crystal violet. The same number of cells were plated into 6-well plates for wound healing assays. When cells were almost 100% confluent, wounds were created using a 200-ul pipet of nearly the same width. The images were captured at the beginning and 24h after the wound formation respectively.

### ER tracker staining assay

After the adherent cells grew to confluency, the culture medium was removed, and the cells were washed with Hanks' Balanced Salt Solution (HBSS). A proper amount of preheated working solution containing the probe was added at 37 °C. Cells were incubated for 15 minutes with the ER-Tracker Red Kit (Beyotime), and subsequently, the working solution was replaced with the culture medium. The images were observed under a confocal microscope. DAPI was used to stain the nuclei.

### Bioinformatics analysis

TRPM2 expression data and the clinical features of ccRCC samples were obtained from the TCGA database (https://xenabrowser.net/) and Oncomine database (Yusenko and Lenburg Renal datasets) (https://www.oncomine.org). The RMS R package constructed the nomogram and the corresponding calibration plots (https://cran.r-project.org/web/packages/rms/). The cBioPortal (http://www.cbioportal.org/) was used to download the co-expression genes data with TRPM2. The DAVID (https://david.ncifcrf.gov/tools.jsp) was used to implement Gene Ontology (GO) and Kyoto Encyclopedia of Genes and Genomes (KEGG) analysis. The GSEA (http://software.broadinstitute.org/gsea/index.jsp) was used to elucidate the signaling pathways involved with TRPM2 (statistical significance: FDR < 25% and P < 0.05). Tumor IMmune Estimation Resource (TIMER, https://cistrome.shinyapps.io/timer/) was employed to examine the association between TRPM2 and immune cell infiltration of CD4+ T cells, CD8 + T cells, B cells, neutrophils, dendritic cells, and macrophages.

### Statistical analysis

GraphPad (Version 6.0) and SPSS (Version 25.0) were employed for data processing. The data between the two groups were analyzed with an unpaired Student's t-test or paired t-test. One-way ANOVA was applied to compare the data from more than two groups. Pearson's chi-squared (χ2) test was applied to analyze the correlation between TRPM2 level and clinicopathological features. Kaplan-Meier (KM) curves were analyzed with a log-rank test. Univariate and multivariate Cox regressions were conducted to analyze the prognostic value of TRPM2. A P-value < 0.05 was considered significant (* P < 0.05, ** P < 0.01, *** P < 0.001, **** P <0.0001).

## Results

### TRPM2 is highly expressed in ccRCC and varies with clinicopathological parameters

**Figure 1:**
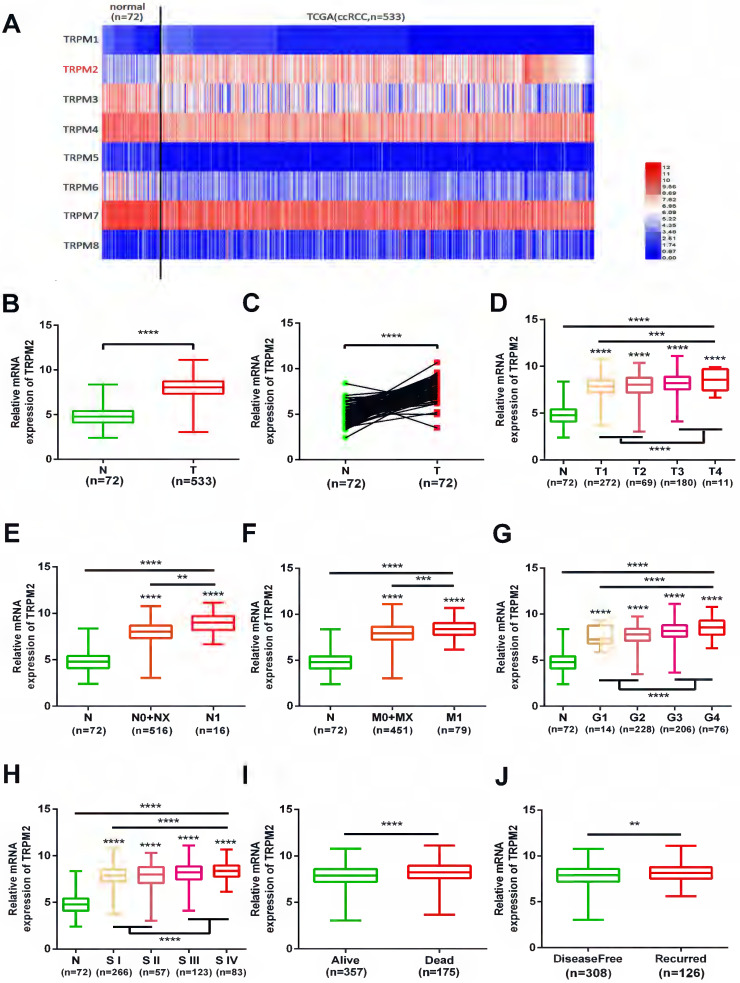
** TRPM2 upregulated in ccRCC and varies with clinicopathological parameters. (A)** Heatmap shows that TRPM2 of TRPMs family is significantly upregulated in ccRCC. **(B-C)** Comparison of TRPM2 expression in paired and non-paired groups from the TCGA-KIRC database. **(D-J)** The TRPM2 expression in ccRCC from the TCGA database was assessed within different clinicopathological parameters: T stage (D), N stage (E), metastasis status (F), Fuhrman grade (G), TNM stage (H), Overall survival status (I), Disease-free status (J). The student's t-test was used to test statistical differences. The data are presented as the means ± SD.

We investigated the expression profile of TRPMs in the KIRC module of the TCGA database, and the heatmap indicated that among TRPM1-8, TRPM2 was significantly upregulated in the tumor samples (Figure 1A). Therefore, we focused on TRPM2 for further analysis. The expression of TRPM2 was higher in the tumor tissues than in normal tissues (Figure 1B), and a similar result was observed in 72 paired tumor and corresponding normal tissues (Figure 1C). Moreover, the higher TRPM2 level was closely related to the higher T stage (T3+T4), N stage (N1), M stage (M1), Fuhrman grade (G3+G4), TNM stage (StageIII+StageIV), worse overall survival (OS) and disease-free status (DFS, Figure 1D-J). Additionally, a chi-square test also verified these results (Table 1). All clinicopathological parameters were significantly associated with TRPM2 expression except for age and gender. Therefore, our results indicated that higher TRPM2 levels might be related to worse outcomes in ccRCC.

### TRPM2 is of remarkable prognostic value in ccRCC

**Figure 2:**
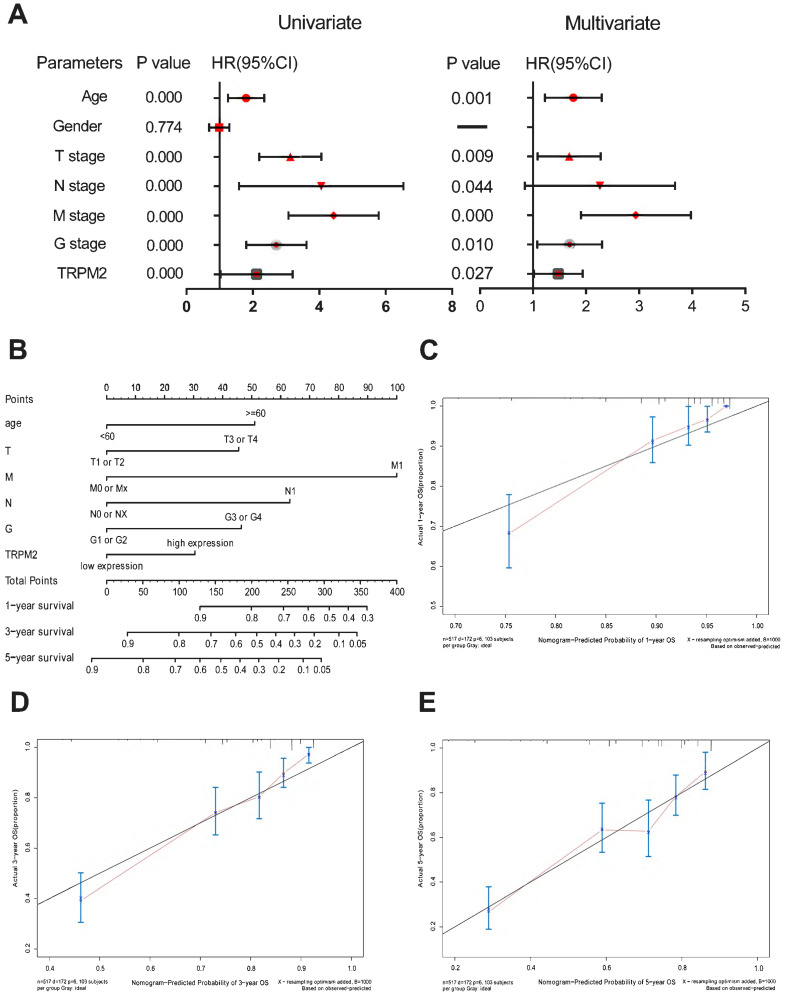
** TRPM2 is of remarkable prognostic value in ccRCC. (A)** Univariate and multivariate Cox regressions presented by a Forest Plot. **(B)** A prognostic nomogram (c-index = 0.754) predicting 1-, 3-, and 5-year overall survival of TCGA-KIRC patients based on different independent prognostic factors including age, T stage, N stage, M stage, Fuhrman grade, and TRPM2 expression level. **(C-E)** Calibration curves of the above nomogram to show the accuracy of nomogram prediction.

First, we divided all available tumor data from KIRC into two subgroups based on the median TRPM2 expression level. Univariate and multivariate Cox regressions were conducted via SPSS 25.0 to examine whether TRPM2 is an independent marker with a prognostic value. The data were presented in Table 2 and the corresponding Forest Plot (Figure 2A). Then, we used the independent prognostic factors, including age, T, N, M stage, Fuhrman Grade, and TRPM2 expression, screened by univariate and multivariate Cox regressions to construct a nomogram to assess 1, 3, and 5-year OS for KIRC patients (Figure 2B), with the C-index at 0.754 (95%CI: 0.681-0.827; P <0.0001). The calibration plots showed that the nomogram-predicted OS was highly consistent with the actual OS (Figure 2C-E). Furthermore, we performed Kaplan-Meier survival analysis on TRPM2 (Supplementary Figure 1). We found that the results were consistent with our previous analysis indicating that higher TRPM2 expression patients had shorter OS (Supplementary Figure 1A). We also conducted the same analysis among different clinicopathological parameters (T1+T2, N0+NX, M1, Stage I + Stage II, Age≤60, age>60, Male, Female, Supplementary Figure 1B-I). However, subgroups including T3+T4, N1, M0, Fuhrman Grade, and Stage III+IV didn't show a statistically significant difference. In summary, TRPM2 can be an independent risk factor of ccRCC.

### TRPM2 can be a diagnostic marker of ccRCC

**Figure 3:**
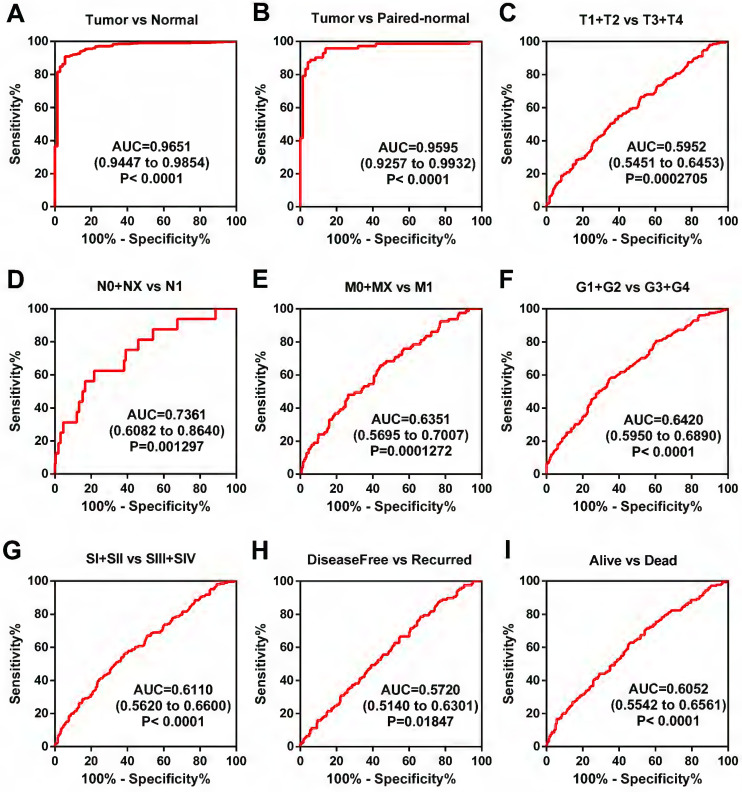
** TRPM2 can be a diagnostic marker of ccRCC. (A-B)** The ROC shows that TRPM2 could significantly differentiate the tumor from unpaired and paired normal tissues. **(C-I)** ROC curve in subgroups: T stage (C), N stage (D), M stage (E), G stage (F), TNM stage (G), Disease-free survival status (H), and Overall survival status (I).

The above results showed that a higher level of TRPM2 is closely associated with worse outcomes in ccRCC, but the diagnostic value of TRPM2 is still unclear. Therefore, ROC (receiver operating characteristic curve) was used to depict the diagnostic value of TRPM2 in various clinicopathological parameters. The results indicated that TRPM2 expression could easily distinguish tumor tissues from normal renal tissues with the area under the curve (AUC) of 0.9651 (95% CI: 0.9447-0.9854; P<0.0001; Figure 3A). Additionally, other subgroups also presented diagnostic values, including cancer vs. para-cancer (Figure 3B), T1+T2 vs. T3+T4 (Figure 3C), N0+NX vs. N1 (Figure 3D), M0+MX vs. M1 (Figure 3E), G1+G2 vs. G3+G4 (Figure 3F), Stage I+II vs. Stage III+IV (Figure 3G), Disease-free vs. Recurred (Figure 3H), and Alive vs. Dead (Figure 3I). AUC, 95% CI, and P-values are presented in the respective figure legends. All these results indicated that TRPM2 exhibits a high diagnostic value in ccRCC.

### The potential function of TRPM2 in cancer revealed by DAVID and GSEA

**Figure 4:**
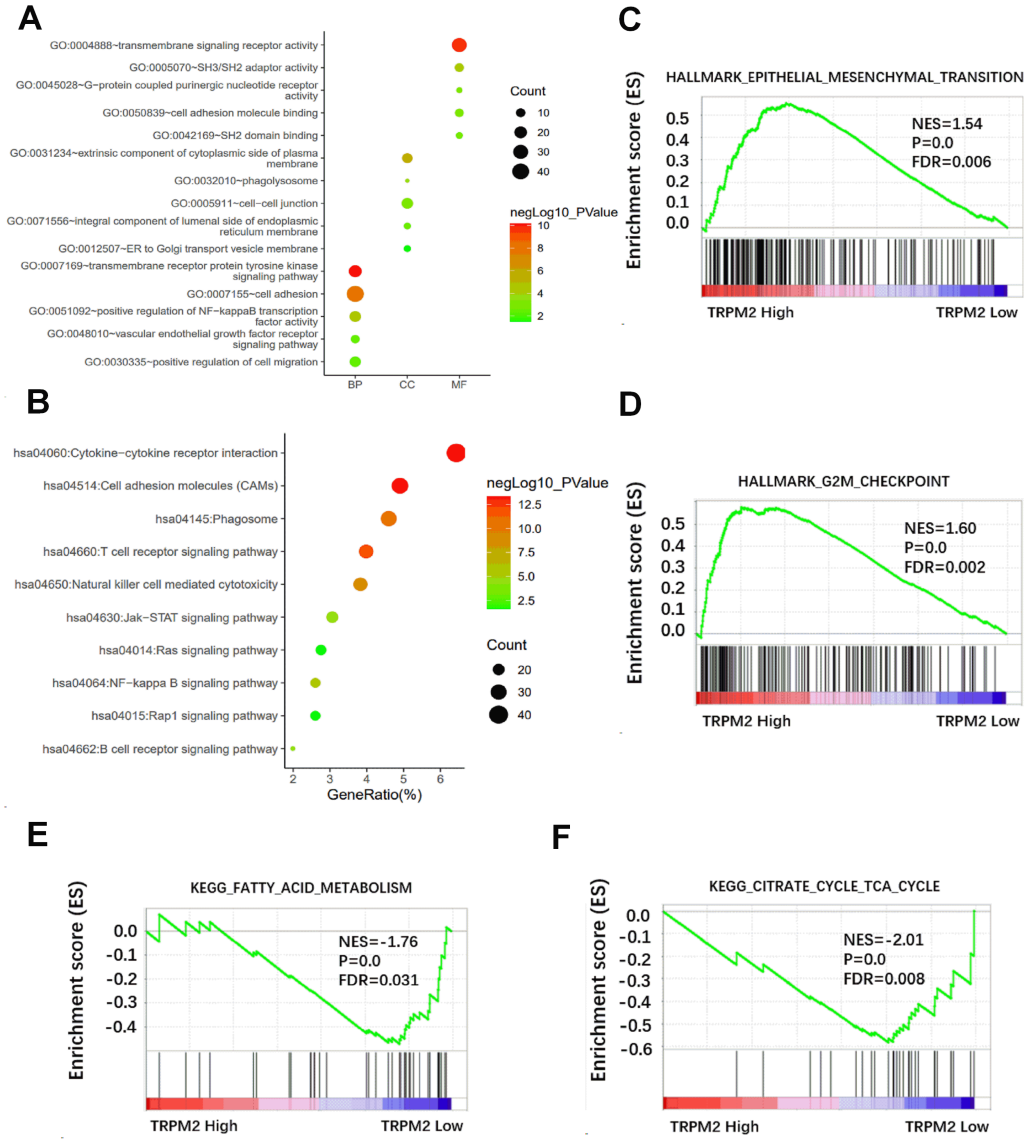
** The potential function of TRPM2 in cancer was revealed by DAVID and GSEA. (A)** GO analysis including biological process (BP), cellular component (CC), molecular function (MF). **(B)** The relative pathway shown by KEGG. **(C-D)** GSEA analysis shows that TRPM2 might be involved in the Hallmarks of epithelial-mesenchymal transition (EMT) and G2M checkpoint. **(E-F)** GSEA showed TRPM2 expression was correlated with KEGG pathways of fatty acid metabolism and citrate cycle.

We first downloaded the co-expression genes data with TRPM2 in KIRC from cBioPortal and selected the genes with r > 0.5 and P < 0.05. Then the selected genes were used to perform GO and KEGG in DAVID, and the results were visualized (Figure 4A, B). Results showed that TRPM2 is related to cell adhesion, NF-kappa B signaling, ER to Golgi transport vesicle, VEGFR signaling, and pathways involved in the immune system. GSEA revealed that TRPM2 was associated with hallmarks of EMT and G2M checkpoint (Figure 4C, D) and KEGG pathways of fatty acid metabolism and citrate cycle (Figure 4E, F).

### TRPM2 facilitates the malignancy of ccRCC *in vitro*

**Figure 5:**
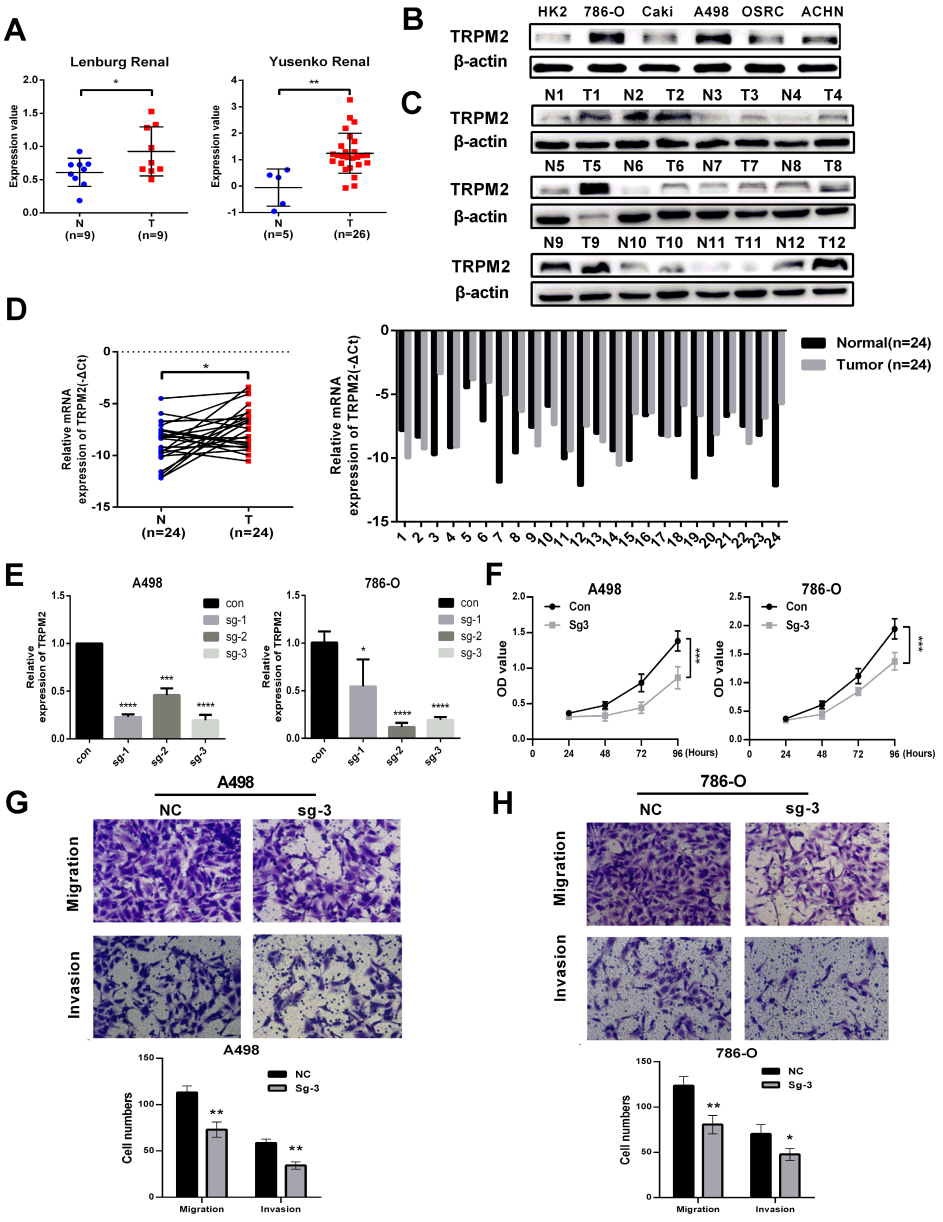
** TRPM2 is upregulated in ccRCC cells and tissues and facilitates the malignancy of ccRCC *in vitro*. (A)** Two cohorts from Oncomine confirmed that TRPM2 was upregulated in ccRCC tissues. **(B-D)** TRPM2 expression was upregulated in ccRCC cell lines (B) and patients' tumor tissues (C). (D) qRT-PCR also indicated that TRPM2 was upregulated in tumor tissues. **(E)** TRPM2 was successfully depleted in A498 and 786-O cells. (F) Knocking out TRPM2 restrained the proliferation of A498 and 786-O cells. **(G-H)** Knocking out TRPM2 significantly hindered the migration and invasion ability of A498 and 786-O cells.

**Figure 6:**
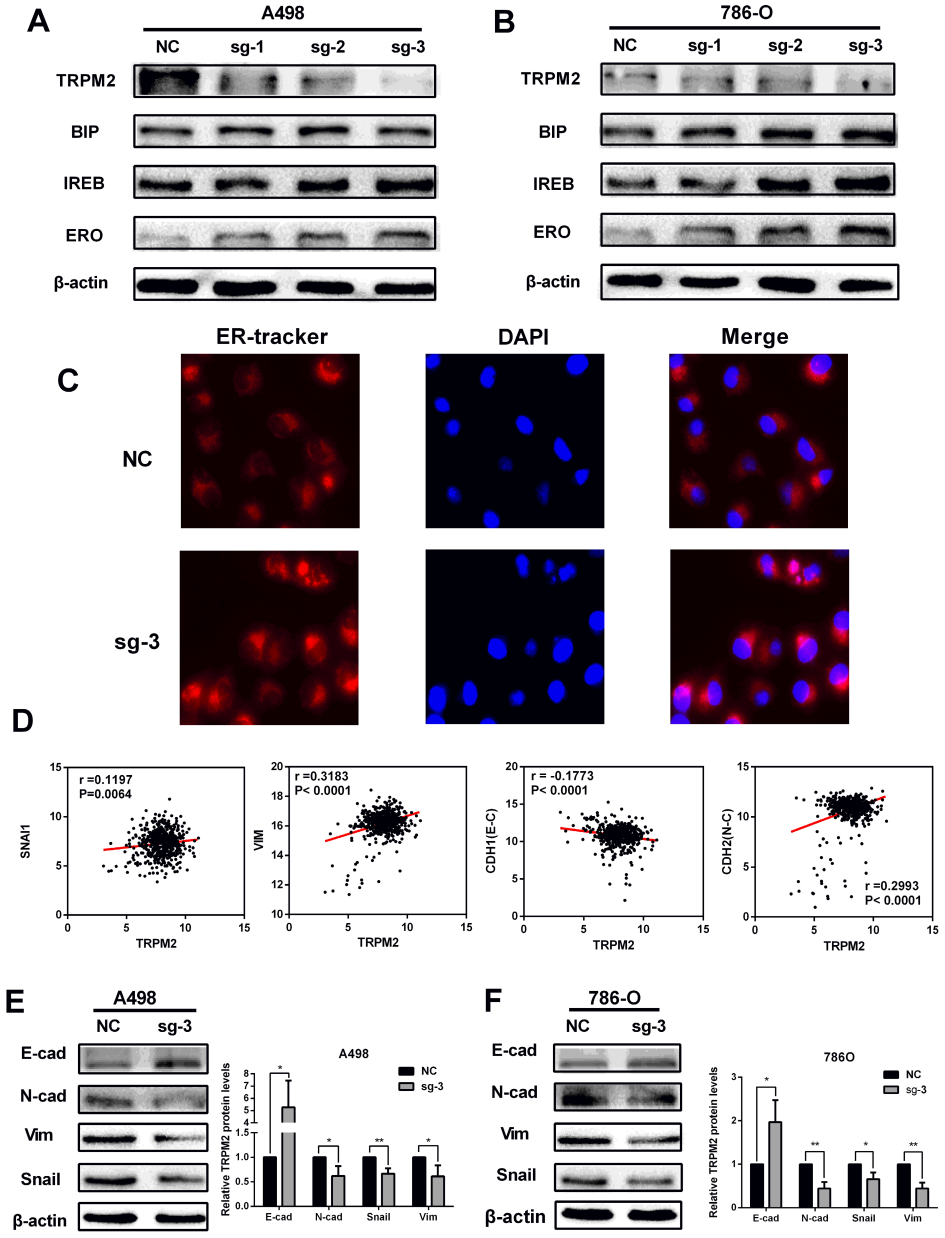
** TRPM2 downregulation represses ccRCC progression by impeding EMT via enhancing ER stress in cancer. (A-B)** Knocking out TRPM2 promotes the expression of ER stress biomarkers. **(C)** ER tracker staining assay indicates ER stress activity is more evident in sg-TRPM2 cells (DAPI stained nucleus). **(D)** The relationship between the expression of TRPM2 and EMT biomarkers. **(E-F)** Western blot shows that inhibiting TRPM2 expression suppresses EMT.

The expression data of TRPM2 from Oncomine also indicated that TRPM2 was more highly expressed in cancer than in normal kidney tissues (Figure 5A). To further verify the above results, we evaluated the mRNA and protein levels of TRPM2 in ccRCC cell lines and tissues, and the results showed an upregulation of TRPM2 both in ccRCC cell lines and tissues compared to the controls (Figure 5B-D). The quantification of western blots in Figure 5C is shown in Supplementary Figure 2A-B. To assess the carcinogenic function of TRPM2, we constructed TRPM2-knockout cell lines with TRPM2 sgRNA plasmids (Figure 5E). After verifying the downregulation achieved by sgRNA using PCR (Figure 5E) and western blotting assays (Figure 6A, B), sg-TRPM2-3 was used for further experiments. CCK-8 and colony formation assays indicated that TRPM2 knock-out inhibited cell proliferation (Figure 5F). The colony formation assays indicated the same results with CCK-8 assays (Supplementary Figure 2C-D). Moreover, transwell assays exhibited a decreased migration and invasion ability of ccRCC cells upon TRPM2 depletion *in vitro* (Figure 5G-H). We also conducted wound healing assays to confirm the effect of TRPM2 silencing on the migration and invasion ability of tumor cells (Supplementary Figure 2E-F). All these functional tests revealed that TRPM2 was upregulated in ccRCC, and its depletion could repress the malignancy of ccRCC cells.

### TRPM2 depletion represses ccRCC progression by impeding EMT via enhancing ER stress in cancer

TRPM2, as in ion channel, mainly participates in calcium metabolism 12 and plays an oncogenic role in several cancers 29. Besides, ER stress is also related to calcium homeostasis 18 and is involved in cancer regulation 22 and cell survival regulation 23. Therefore, we speculated that TRPM2 may play a certain role during ER stress process. Western blotting was performed to determine the relationship between TRPM2 and ER stress. The results showed an upregulation of ER stress biomarkers in the sg-TRPM2 cells compared to those transfected with sgRNA negative control (NC) plasmids (Figure 6 A-B). ER Tracker imaging analysis also showed enhanced ER stress activity in sg-TRPM2 cells (Figure 6C). Additionally, our previous analysis revealed the involvement of TRPM2 in EMT (Figure 4C), so we speculated that TRPM2 affected EMT via ER stress. To verify this hypothesis, firstly, the expression data from TCGA-KIRC was analyzed to examine the correlation between TRPM2 and EMT biomarkers (Figure 6D). Furthermore, the western blotting analyis showed that the protein levels of vimentin (VIM), N-cadherin (CDH2) and SNAIL1 were reduced while E-cadherin (CDH1) was increased upon TRPM2 depletion (Figure 6E-F). All these results revealed that sg-TRPM2 inhibited the proliferation of ccRCC cells. Thus, knock-out of TRPM2 enhances ER stress and suppresses EMT, further inhibiting tumor growth.

### TRPM2 was closely related to immune cell infiltration in the ccRCC microenvironment

**Figure 7:**
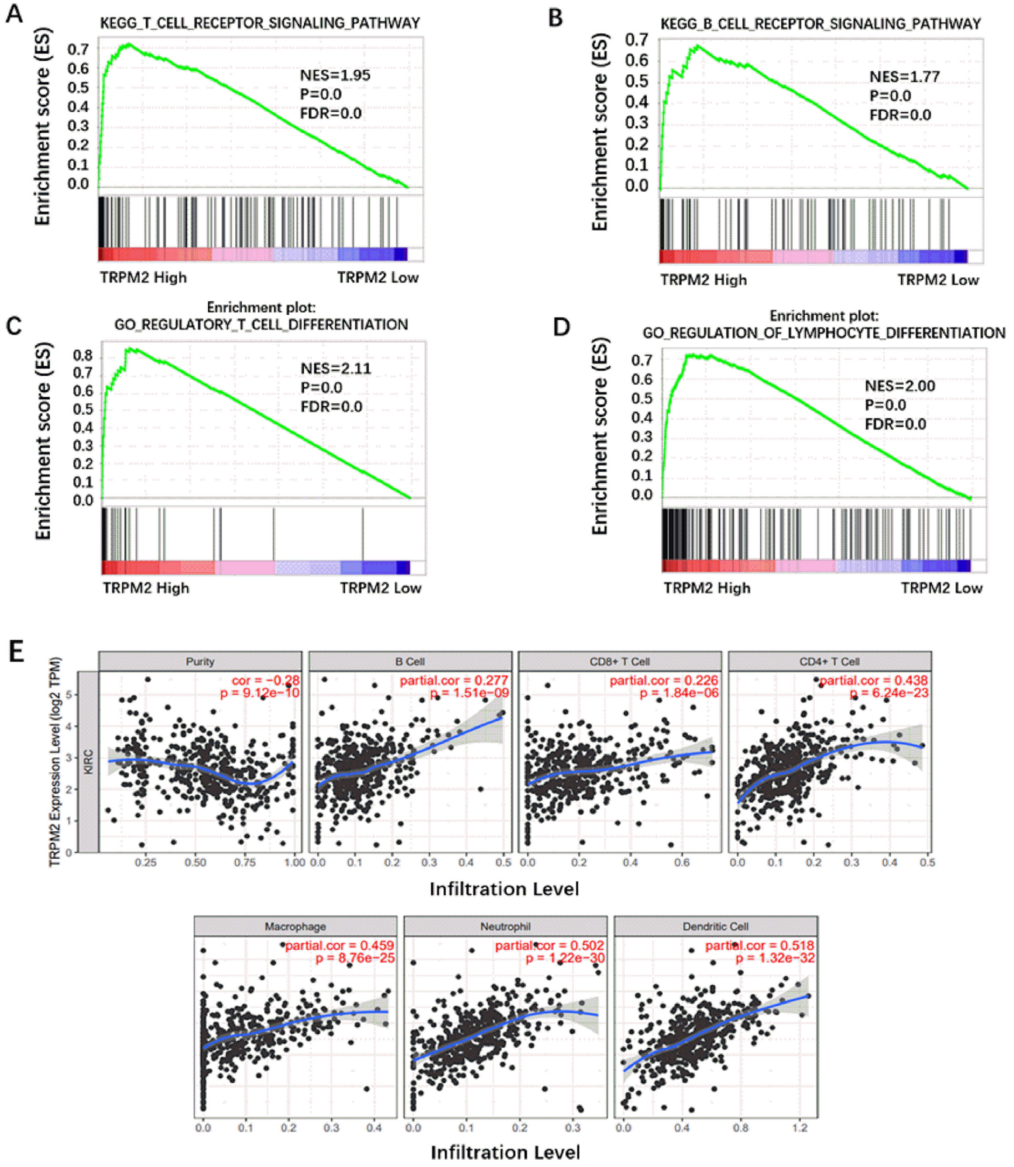
** TRPM2 was closely related to immune cell infiltration in ccRCC. (A-D)** GSEA results show TRPM2 is involved in immune cell regulation. **(E)** Analysis using TIMER indicates that TRPM2 expression is positively associated with immune cell infiltration in ccRCC, including B cell, CD8+ T cell, CD4+ T cell, Macrophage, Neutrophil, Dendritic cell.

Besides being involved in oncogenic pathways, GSEA also indicated that TRPM2 participated in immune cell regulation. As is shown in Figure 7A-D, TRPM2 was involved in the TCR signaling pathway, BCR signaling pathway, regulation of lymphocyte differentiation, and regulatory T cell differentiation. Yasin et al. 30 have shown that ccRCC has the highest immune score among 19 types of cancer. Thus, we used TIMER to investigate the relationship between TRPM2 and immune cell infiltration in ccRCC and showed a close association between them (Figure 7E), indicating a potential immunotherapy target.

## Discussion

RCC consists of various subtypes, among which ccRCC is the most common and causes the most deaths 31. It is a metabolic disease with metabolic variations and lipid accumulation 32. The prognosis of patients with ccRCC has improved remarkably due to more advanced diagnosis and treatment methods such as the Da Vinci surgical system 33 and anti-VEGF or VEGFR therapy 34, 35. However, the mechanisms underlying the tumorigenesis of ccRCC are still unclear. Moreover, the treatment of ccRCC gets more complicated because of drug resistance in these patients. Our work is dedicated to finding a new biomarker for therapy for ccRCC.

The role of the TRPM ion channel (TRPM1-TRPM8) in cancer and other diseases was extensively described before 11, 36, such as in prostate, lung, and pancreatic cancers, but their exact role in ccRCC was rarely studied. TRPM3 promoted tumor growth in ccRCC by regulating oncogenic autophagy through miR-214 37, 38. TRPM2 can protect against tissue damage caused by oxidative stress by stimulating the bioenergetics of mitochondria and by repressing ROS production by transporting Ca2+ into cells 39. Studies have demonstrated that TRPM2 functions in a novel heat-sensitive process 40. But many studies also show that TRPM2 could play a harmful role. Various cancers showed high TRPM2 expression, including gastric 15, prostate 16, lung 17, bladder 41, and breast 42 cancer, suggesting it may play a certain role in carcinogenesis process.

Among all tumor types, ccRCC has the highest correlation with immune cell infiltration 30, 43. Immune checkpoint inhibitors have been applied in clinical immunotherapy for patients with ccRCC. The research on immune infiltration of tumors has attracted people in recent years. Our bioinformatics analysis showed that TRPM2 was correlated with immune cell infiltration in ccRCC, but its precise function and mechanism were still unclear. Knowles et al. reported that TRPM2 plays a critical role in non-specific immunity against Listeria monocytogenes via ROS 44. Interestingly, TRPM2 was also evidently conducive to T cell function via promoting Ca^2+^ influx 45-47. At the same time, C Lory et al. indicated that T cells could function normally without the presence of TRPM2 48. These results might provide a new way to investigate the role TRPM2 might play during adaptive immune cells' reaction to tumors.

In this study, we first investigated the bioinformatics data of TRPM2 in the TCGA-KIRC and Oncomine databases. We found a remarkable upregulation of TRPM2 and a close relation to poor prognosis in ccRCC. Then, by performing KM survival analysis, ROC curve, and univariate and multivariate Cox analyses, we demonstrated that TRPM2 could be an independent diagnostic and prognostic marker of ccRCC. Gene Ontology and GSEA analysis revealed that TRPM2 is involved in oncogenic and metabolic hallmarks and pathways, including EMT, G2M checkpoint, fatty acid metabolism, and citrate cycle. Furthermore, western blotting and qRT-PCR confirmed the results of bioinformatics analysis. Functional and mechanism experiments revealed that TRPM2 promotes ccRCC progression by promoting EMT via reducing ER stress, and knock-out of TRPM2 can reverse the phenotype. Former studies have demonstrated that TRPM2 can decrease ROS production 39 and ROS is positively correlated with ER stress 49; therefore, TRPM2 could promote ccRCC progression by reducing ER stress by decreasing ROS. Our work also illustrated a relationship between TRPM2 and immune infiltration in RCC. However, our study didn't elaborate on how TRPM2 regulates ER stress and how TRPM2 affects ccRCC via immune regulation. Our future study will focus on understanding the precise mechanism involved.

In summary, our study demonstrated that TRPM2 is highly expressed in ccRCC and facilitates tumor growth by inhibiting ER stress and enhancing EMT. Our results indicated TRPM2 as an independent biomarker in ccRCC and offered a new strategy for the treatment of ccRCC.

## Supplementary Material

Supplementary figures.

## Figures and Tables

**Table 1:** Correlation between TRPM2 mRNA expression and clinicopathological parameters of ccRCC patients.

			TRPM2 mRNA expression		
Parameter		Number	Low (n=256)	High (n=261)	P value
Age(years)	<=60	258	127	131	
	>60	259	129	130	0.895
gender	Male	337	158	179	
	Female	180	98	82	0.101
T stage	T1+T2	330	177	153	
	T3+T4	187	79	108	0.013
N stage	N0+ NX	503	254	249	
	N1	14	2	12	0.008
M stage	M0+ MX	439	230	209	
	M1	78	26	52	0.002
G stage	G1+G2	238	146	92	
	G3+G4	279	110	169	0.000
TNM stage	I+II	312	172	140	
	III+IV	205	84	121	0.002

**Table 2:** Univariate and multivariate analysis of TRPM2 mRNA level and patient overall survival.

	Univariate analysis			Multivariate analysis^c^		
Variable	HR^a^	95%CI^b^	P	HR	95% CI	P
Overall survival (n = 517)						
Age (years)						
<=60 (n = 258)						
>60 (n = 259)	1.742	1.281-2.367	0.000	1.704	1.251-2.320	0.001
Gender						
Male (n = 337)						
Female (n = 180)	0.956	0.701-1.303	0.774			
T stage						
T1+T2(n=330)						
T3+T4(n=187)	3.038	2.243-4.114	0.000	1.611	1.127-2.305	0.009
N stage						
N0+NX (n=503)						
N1(n=14)	3.554	1.872-6.747	0.000	1.966	1.017-3.800	0.044
M stage						
M0+MX (n=439)						
M1(n=78)	4.290	3.145-5.854	0.000	2.815	1.967-4.028	0.000
G grade						
G1+G2(n=238)						
G3+G4(n=279)	2.607	1.855-3.662	0.000	1.617	1,121-2.333	0.010
TRPM2						
Low(n=256)						
High(n=261)	1.724	1.270-3.339	0.000	1.429	1.042-1.959	0.027

a Hazard ratio, estimated from Cox proportional hazard regression model.b Confidence interval of the estimated HR.c Multivariate models were adjusted for T, N, M, G classification, age, gender and TRPM2 mRNA level.
