# Supplementary material for: TRPM2 facilitates tumor progression of clear cell renal cell carcinoma by relieving Endoplasmic Reticulum Stress
Source: Int J Med Sci. 2023 Jan 1;20(1):57–69. doi: 10.7150/ijms.77944 (PMC9812806; doi:10.7150/ijms.77944)
Supplement: Supplementary file 1 — Supplementary figures. [file ijmsv20p0057s1.pdf]

TRPM2 facilitates tumor progression of clear cell renal cell carcinoma by relieving Endoplasmic Reticulum Stress

Hongwei Yuan[1#], Weiquan Li[1#], Ning Lou[2#], Tiexi Yu[1], Xiangui Meng[1*], Wen Xiao[1*], Xiaoping Zhang[1*]

[1] Department of Urology, Union Hospital, Tongji Medical College, Huazhong University of Science and Technology, Wuhan 430022, China

[2] Department of Urology, The Central Hospital of Wuhan, Tongji Medical College, Huazhong University of Science and Technology, Wuhan, China

# These authors contributed equally to this study.

*Correspondence author:

Xiaoping Zhang, Department of Urology, Union Hospital, Tongji Medical College, Huazhong University of Science and Technology, Wuhan, 430022, China; E-mail: xzhang@hust.edu.cn

Wen Xiao, Department of Urology, Union Hospital, Tongji Medical College, Huazhong University of Science and Technology, Wuhan, 430022, China; E-mail: xiaowenx11@163.com

Xiangui Meng, Department of Urology, Union Hospital, Tongji Medical College, Huazhong University of Science and Technology, Wuhan, 430022, China; E-mail: 2576452512@qq.com

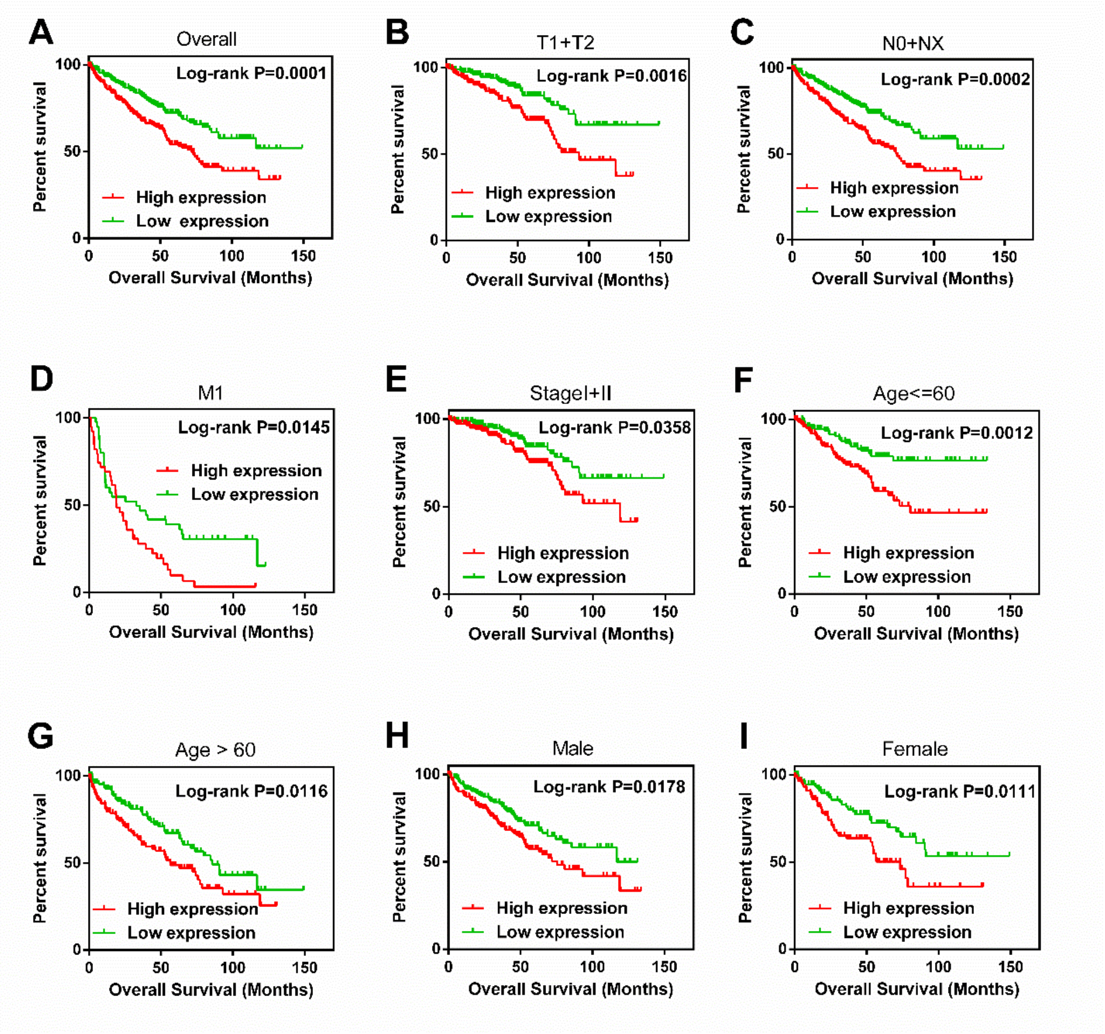

Supplementary Figure 1. Kaplan-Meier overall survival curves of KIRC patients on TRPM2. (A) Patients from the TCGA-KIRC database were divided into two groups based on the median expression value of TRPM2. The survival rate of two corresponding groups was evaluated with the Kaplan Meier method and tested by using a log-rank test. (B-I) Subgroups of ccRCC patients based on clinicopathological parameters were compared with TRPM2 mRNA to overall survival analysis: (B) T1+T2, (C) N0+NX stage, (D) metastasis, (E) TNM I+II stage, (F) Age ≤ 60 years, (G) Age > 60 years, (H) male, (I) female.

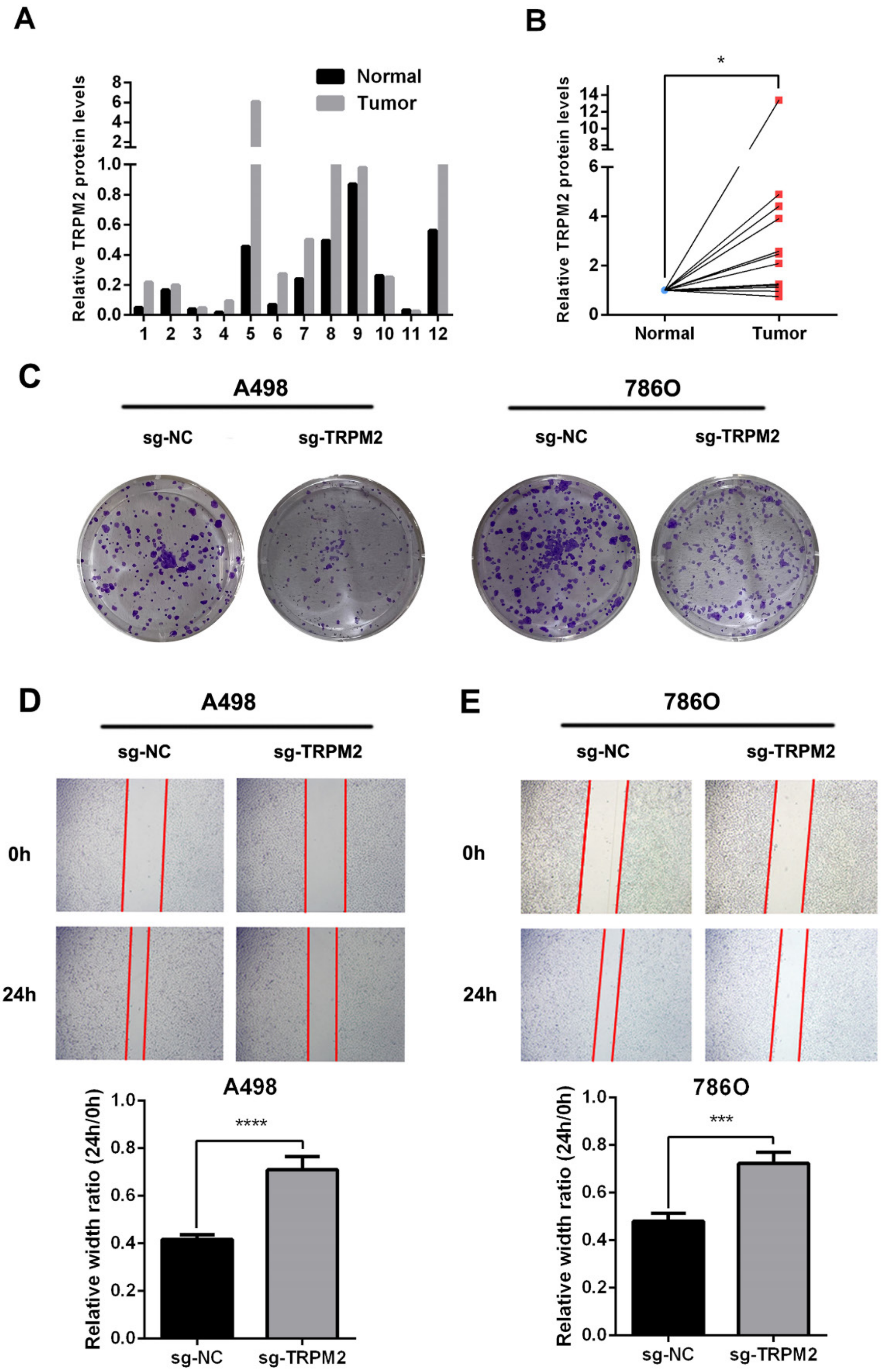

Supplementary Figure 2. (A) The quantification of Western blots in Figure 5C. (B) The paired Student's t test between normal and tumor of data in Figure S2A (Normalized by normal tissue group). (C) The colony formation assays showed TRPM2 silencing impeded the proliferation capacity of ccRCC cells. (D-E) The wound healing assays indicated TRPM2 depletion decreased the migration ability of ccRCC cells. The assays were independently conducted in triplicate.
